# Cardiovascular Aging

**DOI:** 10.1093/geroni/igaf122.665

**Published:** 2025-12-31

## Abstract

In this session you will gain insights into the impact of cardiovascular aging and disease specific monitoring opportunities.

